# Supplementary figures and images for: Uncovering the BIN1-SH3 interactome underpinning centronuclear myopathy
Source: eLife. 2024 Jul 12;13:RP95397. doi: 10.7554/eLife.95397 (PMC11245310; doi:10.7554/eLife.95397)

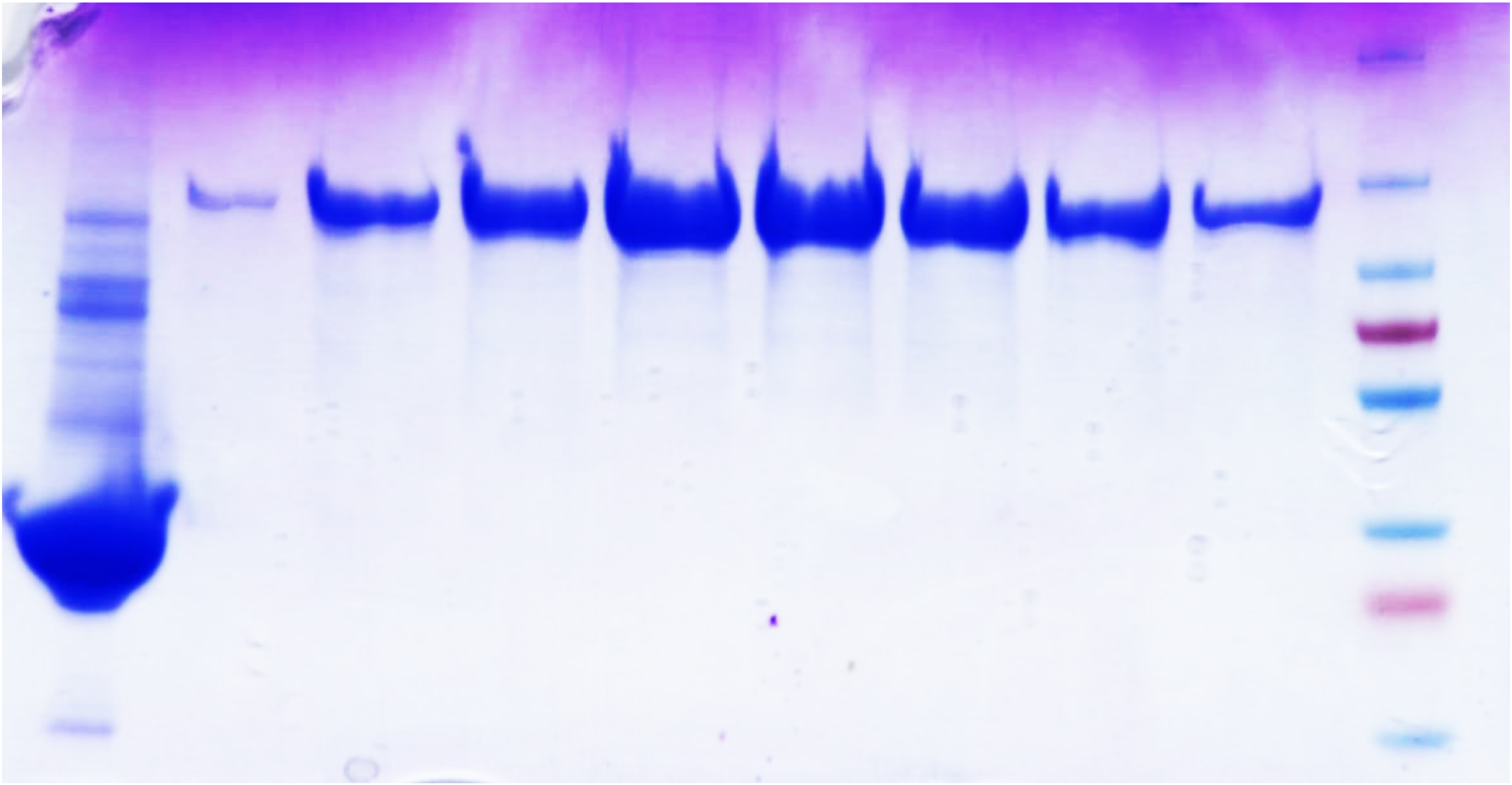

Supplement: Figure 3—figure supplement 1—source data 1. [file elife-95397-fig3-figsupp1-data1.zip › Figure3-figuresupplement1-sourcedata1.png]

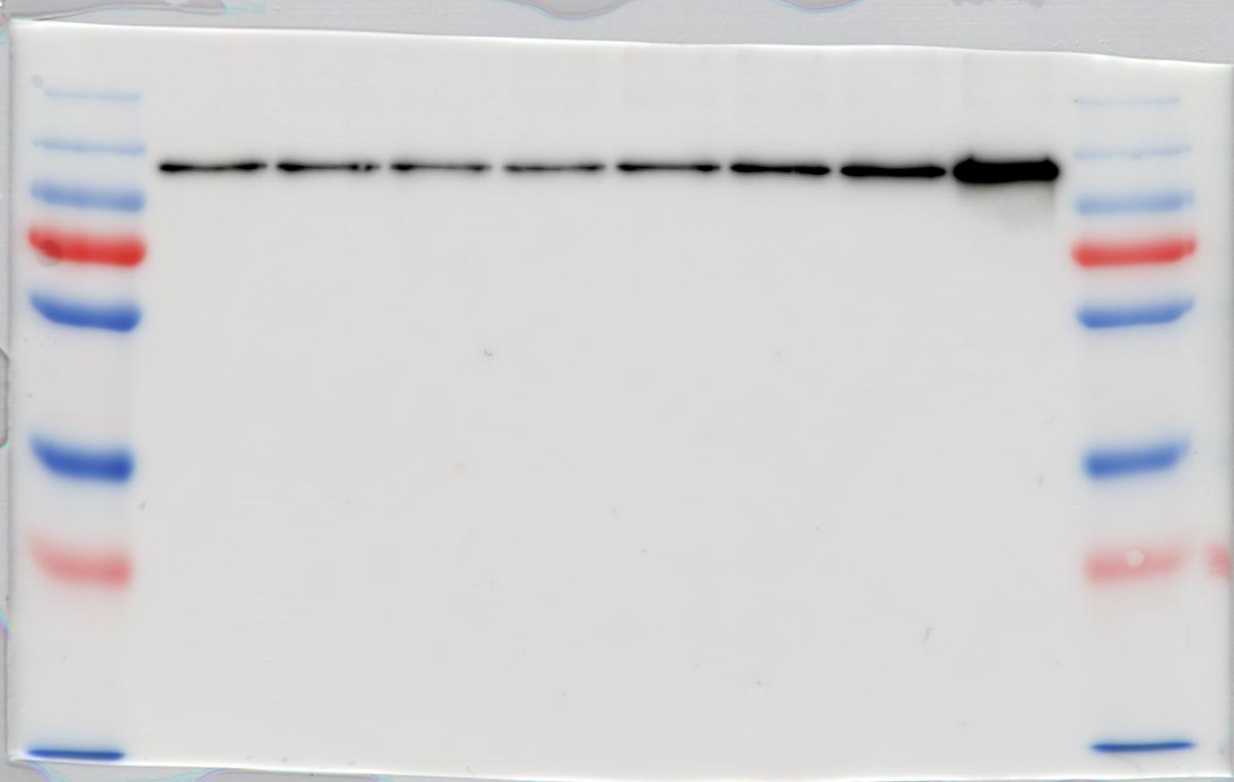

Supplement: Figure 3—figure supplement 2—source data 1. [file elife-95397-fig3-figsupp2-data1.zip › Figure3-figuresupplement2-sourcedata1.png]

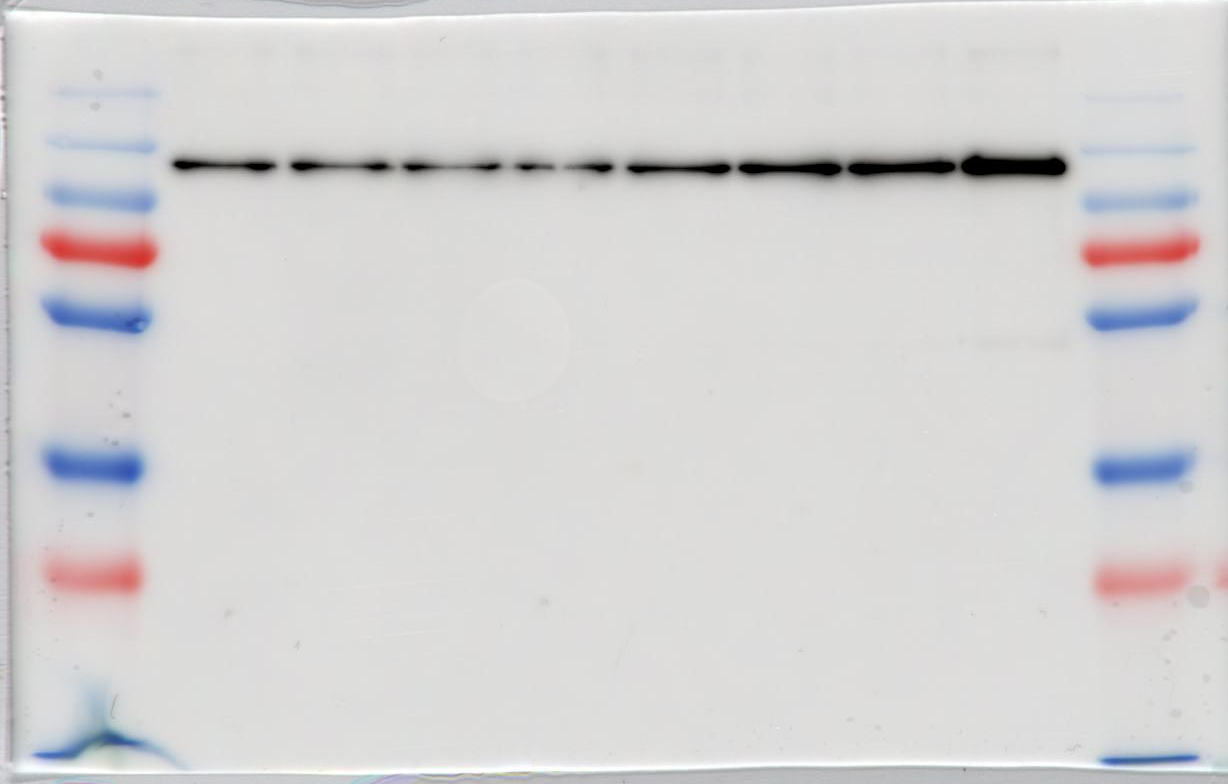

Supplement: Figure 3—figure supplement 2—source data 2. [file elife-95397-fig3-figsupp2-data2.zip › Figure3-figuresupplement2-sourcedata2.png]

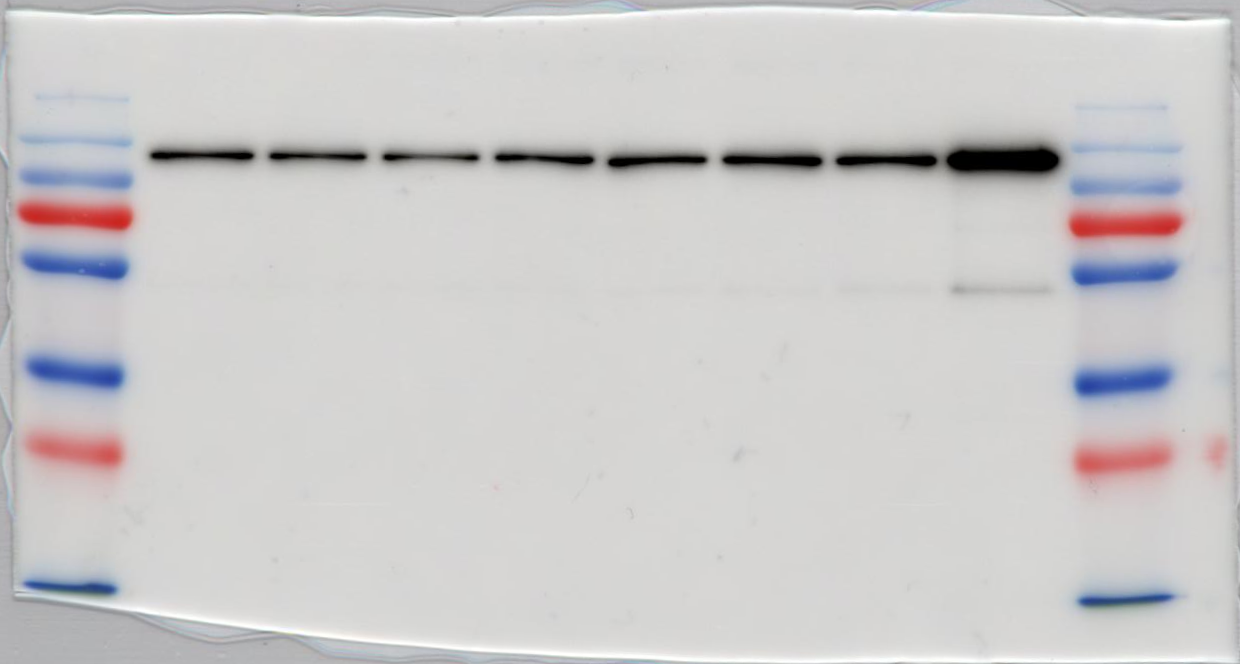

Supplement: Figure 3—figure supplement 2—source data 3. [file elife-95397-fig3-figsupp2-data3.zip › Figure3-figuresupplement2-sourcedata3.png]

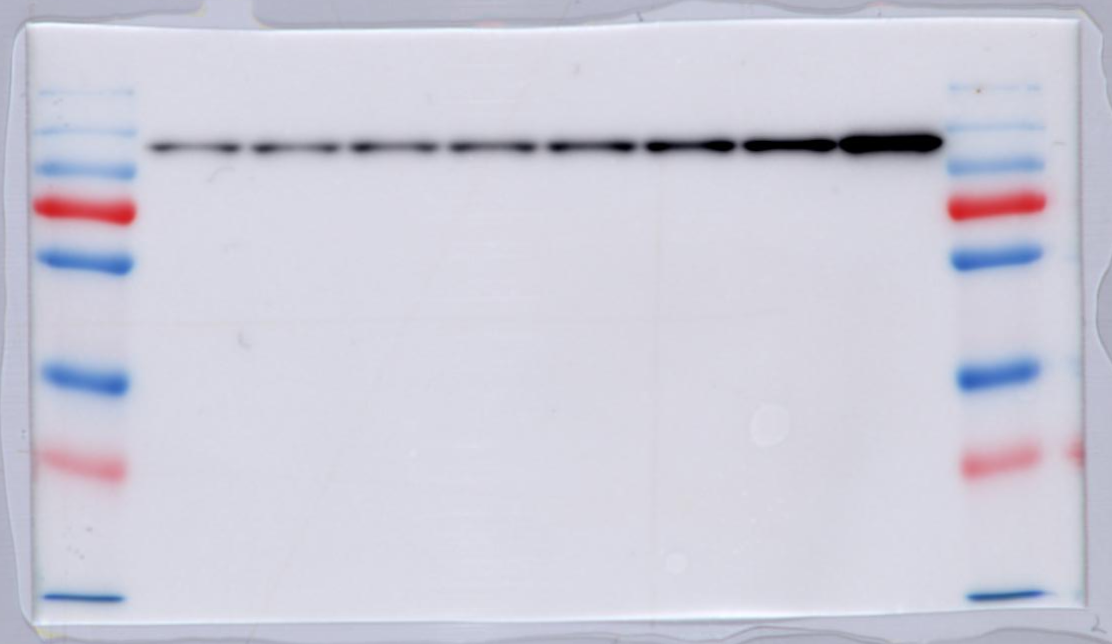

Supplement: Figure 3—figure supplement 2—source data 4. [file elife-95397-fig3-figsupp2-data4.zip › Figure3-figuresupplement2-sourcedata4.png]

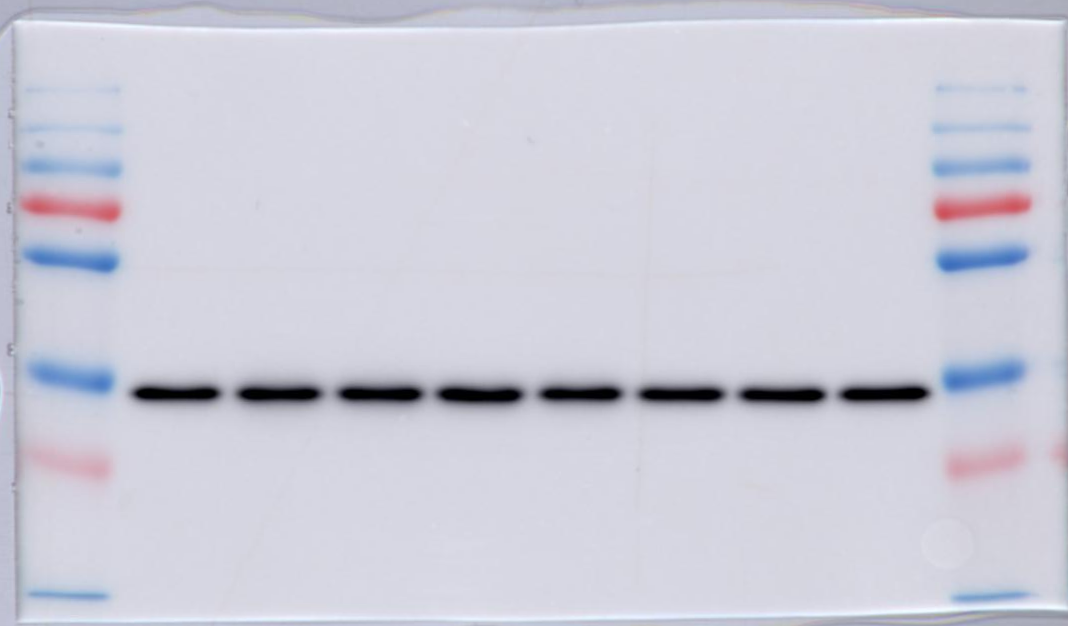

Supplement: Figure 3—figure supplement 2—source data 5. [file elife-95397-fig3-figsupp2-data5.zip › Figure3-figuresupplement2-sourcedata5.png]

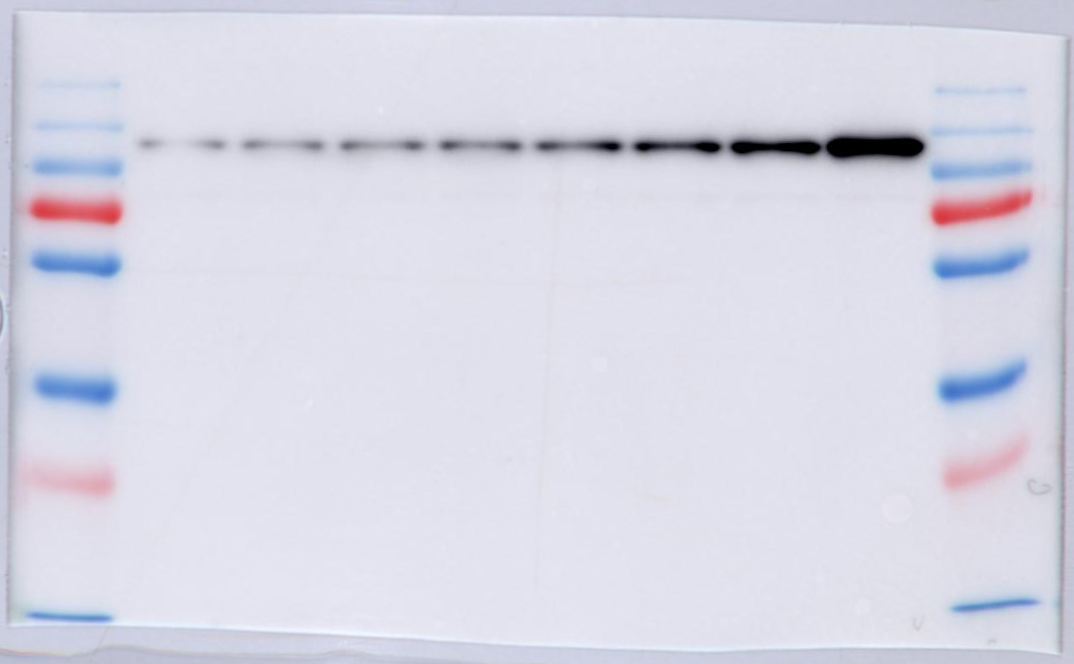

Supplement: Figure 3—figure supplement 2—source data 6. [file elife-95397-fig3-figsupp2-data6.zip › Figure3-figuresupplement2-sourcedata6.png]

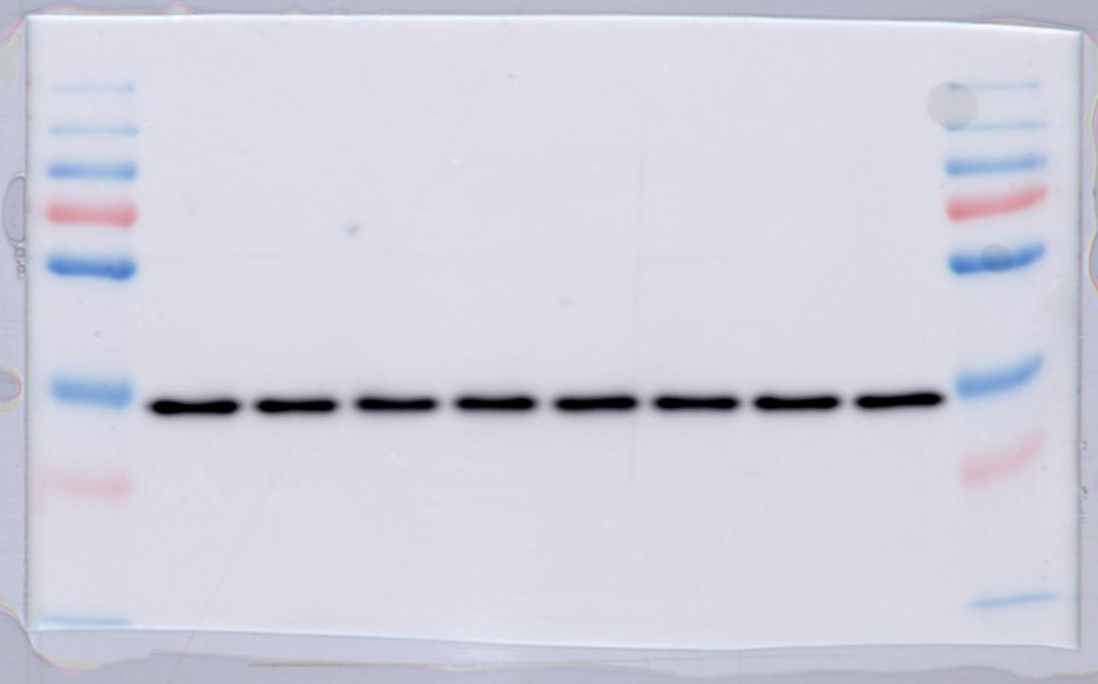

Supplement: Figure 3—figure supplement 2—source data 7. [file elife-95397-fig3-figsupp2-data7.zip › Figure3-figuresupplement2-sourcedata7.png]

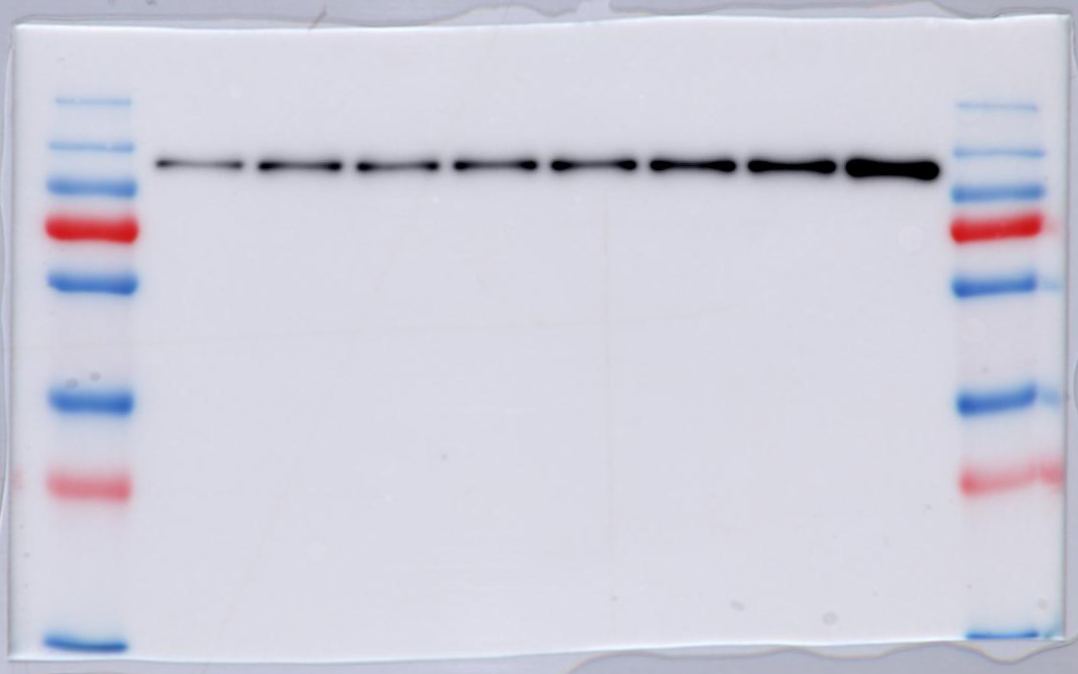

Supplement: Figure 3—figure supplement 2—source data 8. [file elife-95397-fig3-figsupp2-data8.zip › Figure3-figuresupplement2-sourcedata8.png]

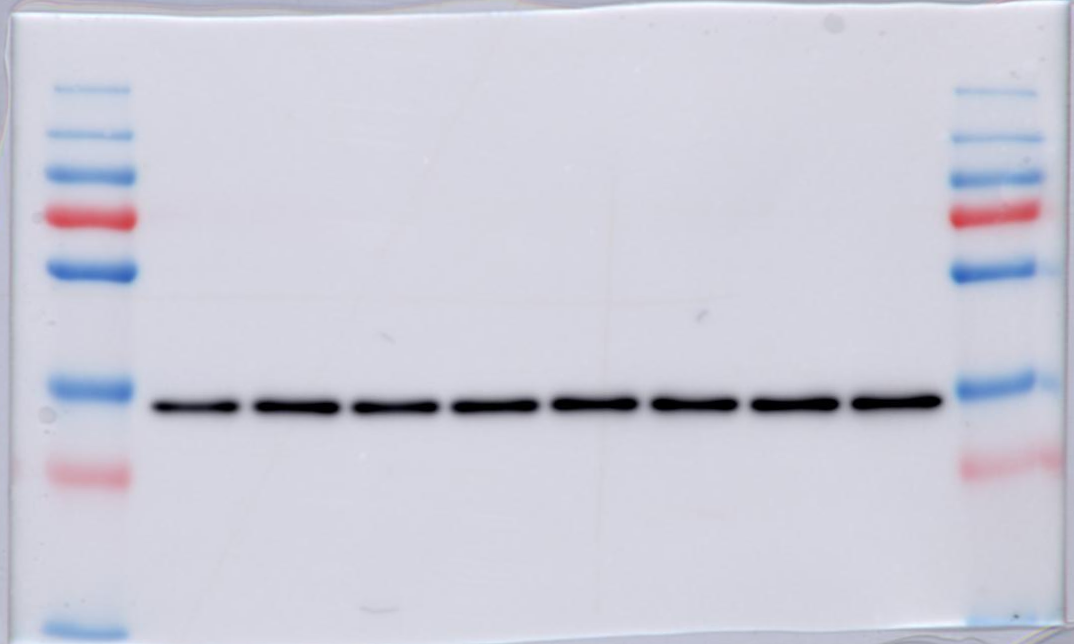

Supplement: Figure 3—figure supplement 2—source data 9. [file elife-95397-fig3-figsupp2-data9.zip › Figure3-figuresupplement2-sourcedata9.png]

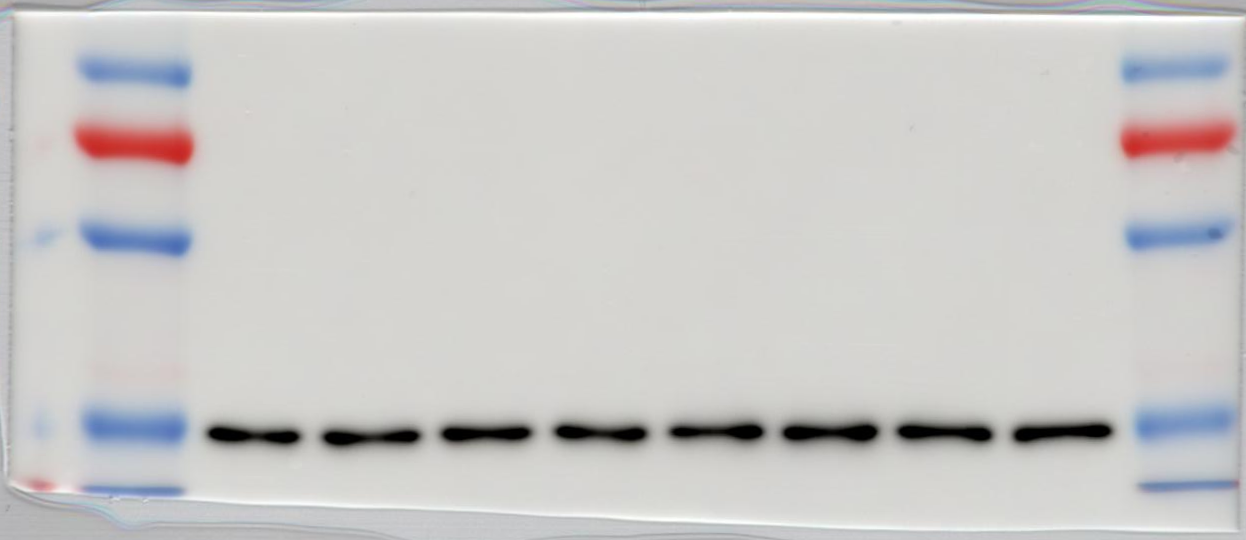

Supplement: Figure 3—figure supplement 2—source data 10. [file elife-95397-fig3-figsupp2-data10.zip › Figure3-figuresupplement2-sourcedata10.png]

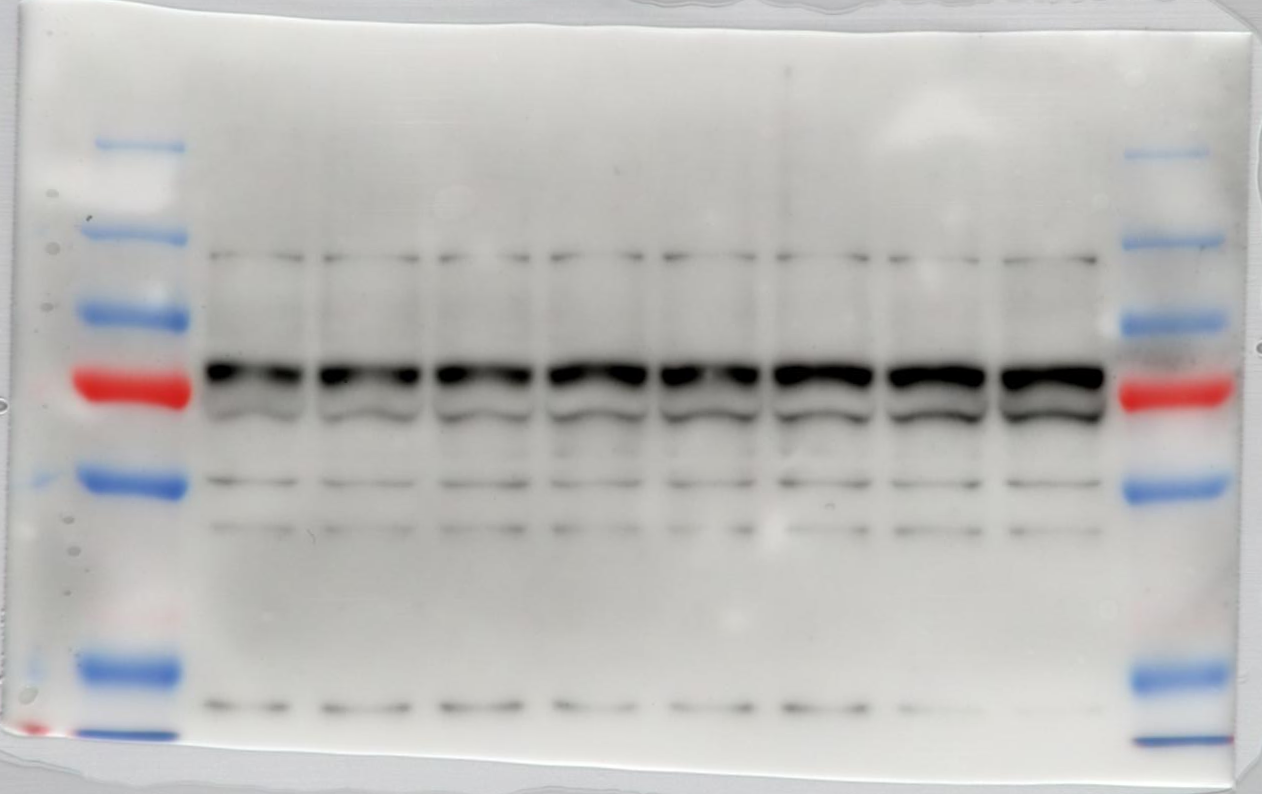

Supplement: Figure 3—figure supplement 2—source data 11. [file elife-95397-fig3-figsupp2-data11.zip › Figure3-figuresupplement2-sourcedata11.png]

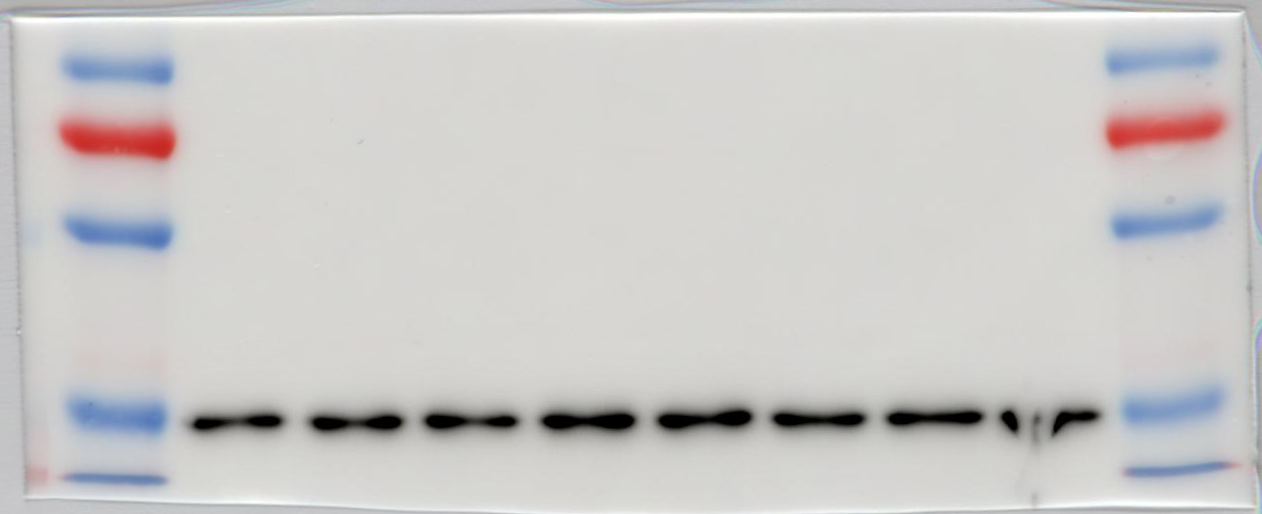

Supplement: Figure 3—figure supplement 2—source data 12. [file elife-95397-fig3-figsupp2-data12.zip › Figure3-figuresupplement2-sourcedata12.png]

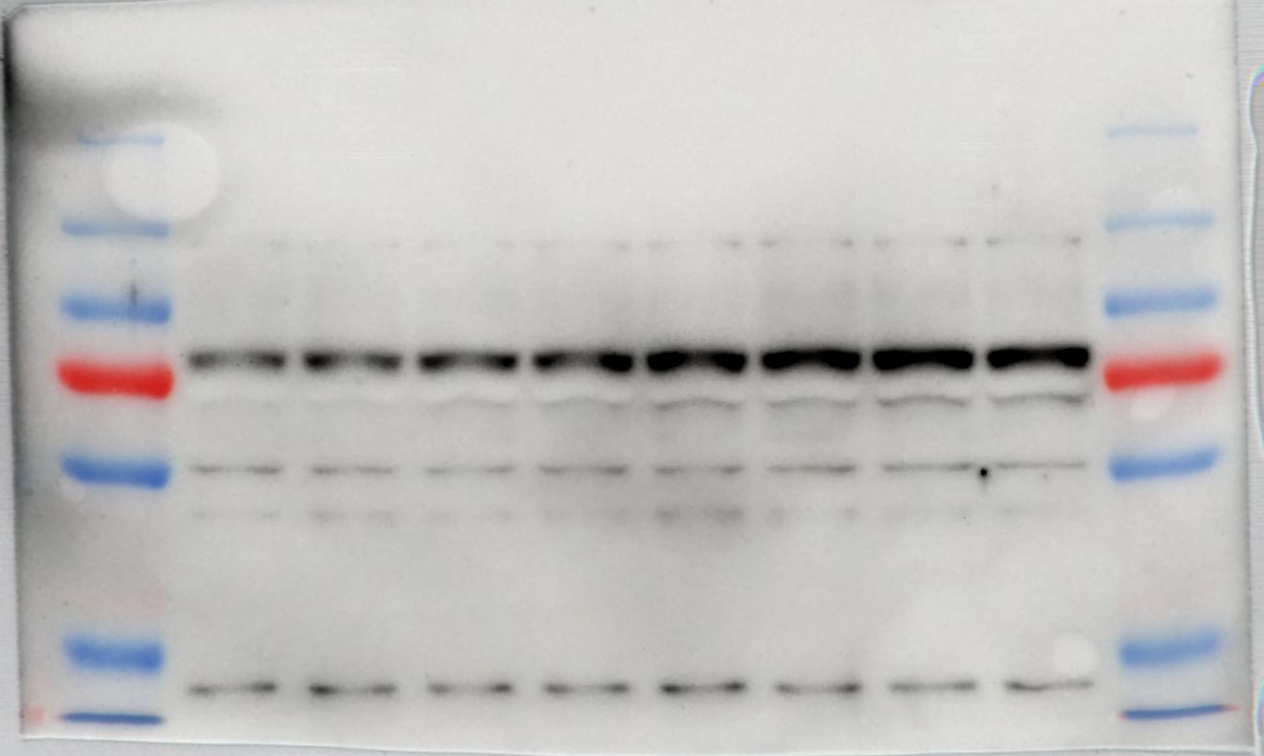

Supplement: Figure 3—figure supplement 2—source data 13. [file elife-95397-fig3-figsupp2-data13.zip › Figure3-figuresupplement2-sourcedata13.png]

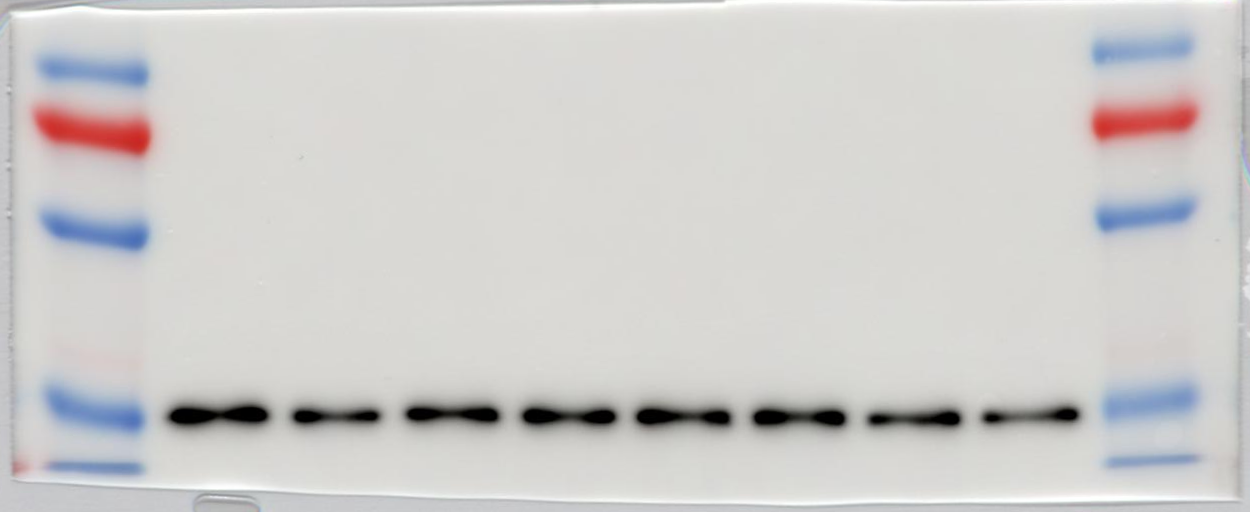

Supplement: Figure 3—figure supplement 2—source data 14. [file elife-95397-fig3-figsupp2-data14.zip › Figure3-figuresupplement2-sourcedata14.png]

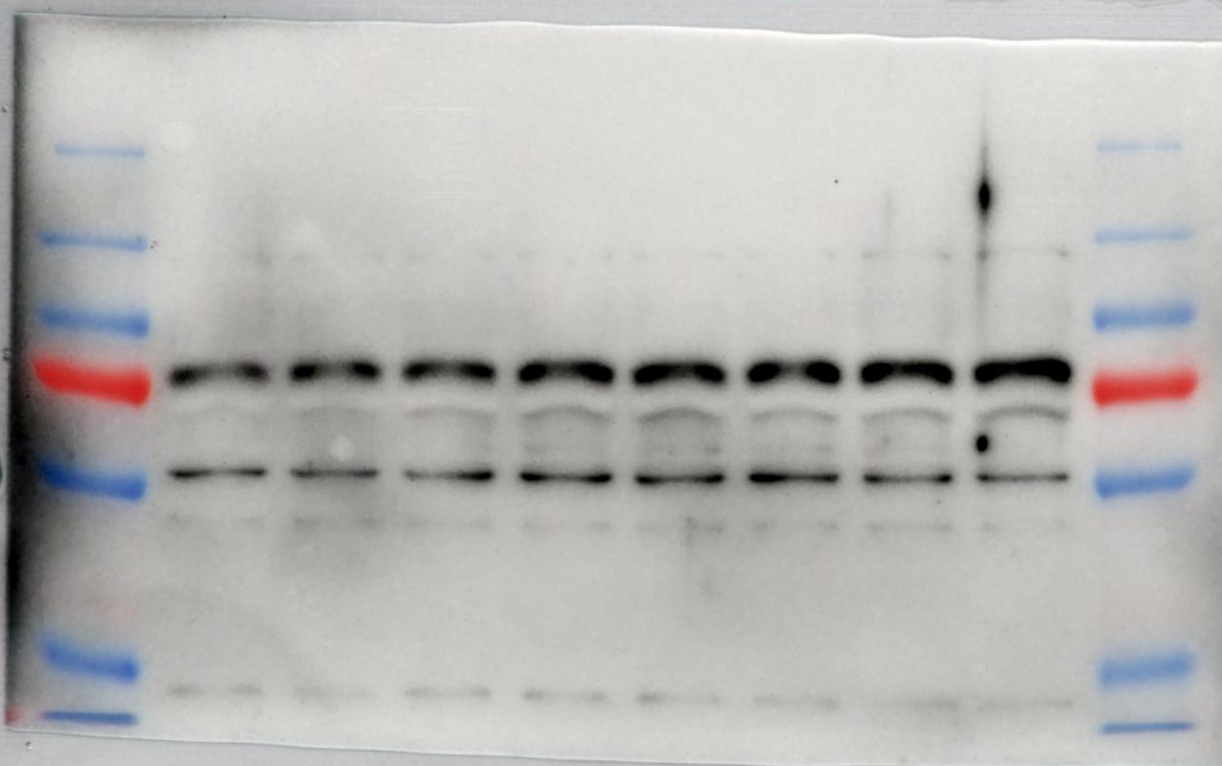

Supplement: Figure 3—figure supplement 2—source data 15. [file elife-95397-fig3-figsupp2-data15.zip › Figure3-figuresupplement2-sourcedata15.png]

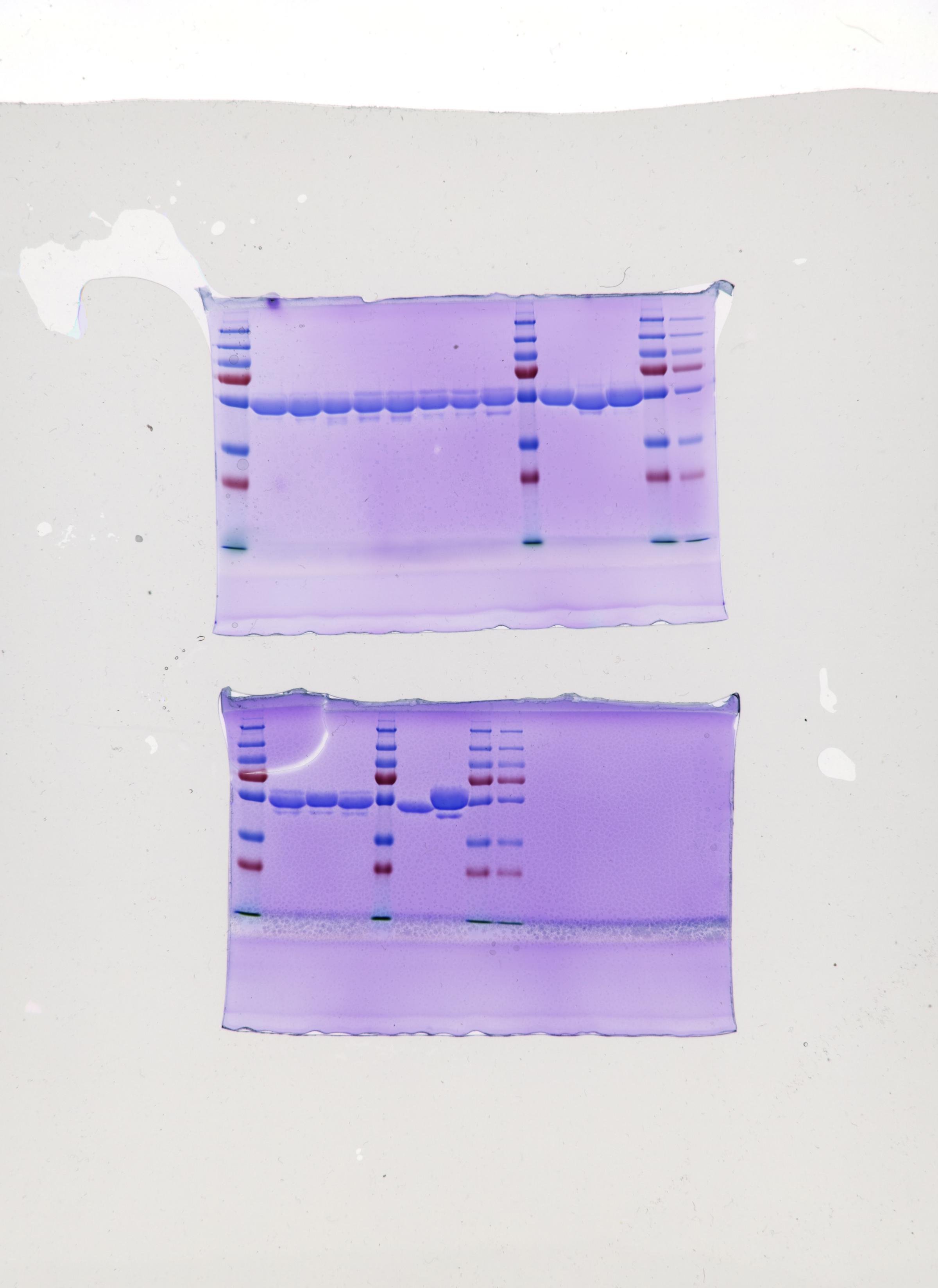

Supplement: Figure 5—figure supplement 2—source data 1. [file elife-95397-fig5-figsupp2-data1.zip › Figure5-figuresupplement2-sourcedata1.jpg]
